# An Effective Hyperspectral Image Classification Network Based on Multi-Head Self-Attention and Spectral-Coordinate Attention

**DOI:** 10.3390/jimaging9070141

**Published:** 2023-07-10

**Authors:** Minghua Zhang, Yuxia Duan, Wei Song, Haibin Mei, Qi He

**Affiliations:** College of Information Technology, Shanghai Ocean University, Shanghai 201306, China; mhzhang@shou.edu.cn (M.Z.); m210911542@st.shou.edu.cn (Y.D.); wsong@shou.edu.cn (W.S.); qihe@shou.edu.cn (Q.H.)

**Keywords:** hyperspectral image, image classification, deep learning, spectral-coordinate attention, long-range dependency

## Abstract

In hyperspectral image (HSI) classification, convolutional neural networks (CNNs) have been widely employed and achieved promising performance. However, CNN-based methods face difficulties in achieving both accurate and efficient HSI classification due to their limited receptive fields and deep architectures. To alleviate these limitations, we propose an effective HSI classification network based on multi-head self-attention and spectral-coordinate attention (MSSCA). Specifically, we first reduce the redundant spectral information of HSI by using a point-wise convolution network (PCN) to enhance discriminability and robustness of the network. Then, we capture long-range dependencies among HSI pixels by introducing a modified multi-head self-attention (M-MHSA) model, which applies a down-sampling operation to alleviate the computing burden caused by the dot-product operation of MHSA. Furthermore, to enhance the performance of the proposed method, we introduce a lightweight spectral-coordinate attention fusion module. This module combines spectral attention (SA) and coordinate attention (CA) to enable the network to better weight the importance of useful bands and more accurately localize target objects. Importantly, our method achieves these improvements without increasing the complexity or computational cost of the network. To demonstrate the effectiveness of our proposed method, experiments were conducted on three classic HSI datasets: Indian Pines (IP), Pavia University (PU), and Salinas. The results show that our proposed method is highly competitive in terms of both efficiency and accuracy when compared to existing methods.

## 1. Introduction

Hyperspectral image (HSI) classification is a hot topic in the field of remote sensing. HSIs, captured by airborne visible/infrared imaging spectrometer (AVIRIS), provide rich spectral and spatial information that is highly valuable for the fine segmentation and identification of ground objects. Therefore, HSIs have been widely applied in various fields such as geological exploration, military investigation, environmental monitoring, and precision agriculture [1,2,3,4].

In the past decades, traditional feature extraction methods for HSI classification, such as k-nearest neighbor [5], random forest [6], Markov random fields [7], and support vector machines (SVM) [8], have been widely used. However, these methods require manual labeling and expert experience, which make them expensive and limited in their ability to extract high-level features. Additionally, HSIs with redundant information also pose challenges for classifiers.

Deep learning methods have received significant attention for their ability to automatically learn robust features from training samples. These methods have been successfully applied to HSI classification, including stacked autoencoder (SAE) [9], recurrent neural network (RNN) [10], deep belief network (DBN) [11], CNNs [12,13,14], and others. These approaches have achieved remarkable results when compared to traditional methods. Chen et al. [15] first introduced deep learning into hyperspectral data and used SAE to obtain spectral and spatial features, respectively, to achieve classification. Later, Hu et al. [16] used five 1×1 convolution layers to capture spectral information for HSI classification but ignored the importance of spatial information. Ying et al. [17] proposed a 3-D convolutional neural network to extract spectral-spatial features of 3-D hyperspectral images to achieve accurate classification of HSI. Yang et al. [18] proposed a two-channel deep convolutional neural network model (TCCNN) to extract joint spectral-spatial features of HSIs and two branches were used to extract spectral and spatial features, respectively. Chen et al. [19] added 3-D convolution to extract spectral-spatial features of HSI based on TCCNN, and the results show that the method is effective. However, 3-D CNN will cause excessive training parameters and high calculation costs. As neural networks become deeper, the extracted features become more abstract and robust. However, the limited number of training samples can lead to overfitting. To address this problem, Zhong et al. [20] and Wang et al. [21] used residual connections [22] and dense connections [23], respectively, to enhance the robustness of the network and avoid overfitting. Due to the ability to perform convolutions on arbitrary graph structures, graph convolutional networks (GCNs) have been applied to HSI. Qin et al. [24] proposed a semi-supervised GCN method, which can flexibly encode irregular non-Euclidean data and effectively express the relationship between each node. However, it requires a large amount of computing cost to construct an adaptive graph structure.

Attention mechanisms [25] have gained attention in the field of vision for their ability to focus on important information and disregard redundant information. The transformer model uses a multi-head self-attention (MHSA) module to capture long-range dependencies in input sequences. Song et al. [26] proposed a hierarchical transformer network that uses MHSA to better extract spectral-spatial information. However, the computational cost of MHSA is high due to the excessive dot-product operations involved. T et al. [27] combined the Squeeze-and-Excitation (SE) Network, known for its effectiveness in channel attention, with CNN for HSI classification, effectively utilizing spectral information. Similarly, Sun et al. [28] proposed a spectral-spatial attention mechanism, adding spectral and spatial attention to each traditional convolution, enabling a higher focus on useful information and improving the classification accuracy. Li et al. [29] proposed a double-branch dual-attention network (DBDA) to capture spectral and spatial features separately, to achieve refinement and optimization of the extracted features. And the coordinate attention network (CA) [30] was proposed to address the high computational cost and complexity of the attention mechanism. It retains spatial coordinate position information and captures global information of image pixels.

Although the above methods have already achieved promising results, they are still facing some problems. (1) The classification performance of CNN-based methods for HSI classification is limited by the size of the convolutional kernels, and it is difficult to capture long-range dependencies between pixels in HSI. (2) HSI typically contains hundreds of continuous spectral bands, but not all bands contribute equally to classification accuracy. The invalid bands not only increase computational cost but also degrade classification performance. (3) Existing methods for HSI classification have complex network architectures, which can lead to inefficient classification results.

Inspired by the attention mechanism, this paper proposes an effective HSI classification network based on MHSA and spectral-coordinate attention. The proposed method first uses a point-wise convolution network (PCN) to remove redundant spectral band information and provide more discriminative features. Then, an M-MHSA module is introduced, which down-samples the *k* and *v* projections to a low-dimensional embedding to alleviate the computing burden caused by dot-product operations in MHSA. The method also assigns weights based on pixel correlation to capture long-range dependencies among the HSI pixels, addressing the limitations of CNNs having a small receptive field. Furthermore, a lightweight spectral-coordinate attention fusion network is proposed. On the one hand, spectral attention is used to model the importance of each spectral feature and suppress invalid channels. On the other hand, the coordinate attention network is used to aggregate features along two spatial directions, which addresses the limitation of MHSA ignoring inherent position information and strengthens the connection between channels. Finally, we conducted experiments on three classical datasets, Indian Pines (IP), Pavia University (PU), and Salinas. The experimental results demonstrate that our proposed method is highly competitive among existing HSI classification methods.

The rest of this paper is organized as follows: the proposed method is described in Section 2. The experiments and analysis are presented in Section 3. The conclusion is drawn in Section 4.

## 2. Proposed Methods

**Figure 1:**
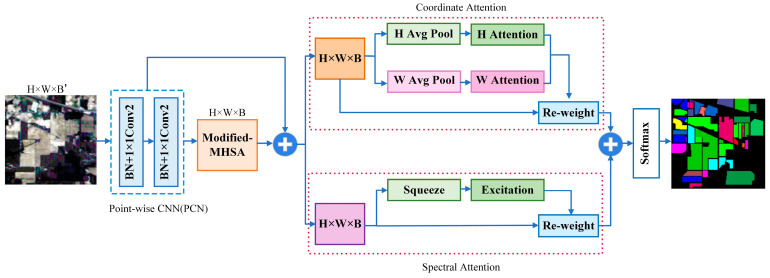
The overall network architecture of the proposed MSSCA.

The goal of HSI classification is to assign a specific label to each pixel in order to represent a particular category. In this paper, we propose an effective network based on multi-head self-attention and spectral-coordinate attention (MSSCA). The overall architecture of the proposed network is depicted in Figure 1.

### 2.1. Point-Wise Convolution Network (PCN)

HSIs often contain redundant bands, which not only increase computational complexity but also negatively impact classification accuracy. To reduce the redundant information and provide more discriminant features for subsequent networks, we propose the PCN to process the band information of the HSI. Specifically, let $X\in R^{H\times W\times B\prime }$ as the HSI input, and the PCN is composed of two 1×1 convolutional layers. Using this network, the output feature map can be expressed as:

(1) $$X_{j}^{l}=f\left(W_{j}^{l}\cdot {\tilde{X}}^{l-1}+b_{j}^{l}\right)$$

where $X^{l}$ represents the output representation of the feature map of the *l*-th spectral convolution layer, $X_{j}^{l}$ represents the value of the *j*-th output feature channel in the *l*-th layer, ${\tilde{X}}^{l-1}=BN\left(X^{l-1}\right)$ denotes the input feature mapping of the *(l-1)*-th convolution layer after batch normalization, $W_{j}^{l}$ and $b_{j}^{l}$ represent the *j*-th convolutional kernel with the size of 1 × 1 and the bias in the *l*-th layer, respectively, and $f\left(\cdot \right)$ is the activation function. The resulting PCN output is then fed as input to subsequent networks, providing robust and discriminative initial spectral characteristics for these networks.

### 2.2. Modified Multi-Head Self-Attention (M-MHSA)

**Figure 2:**
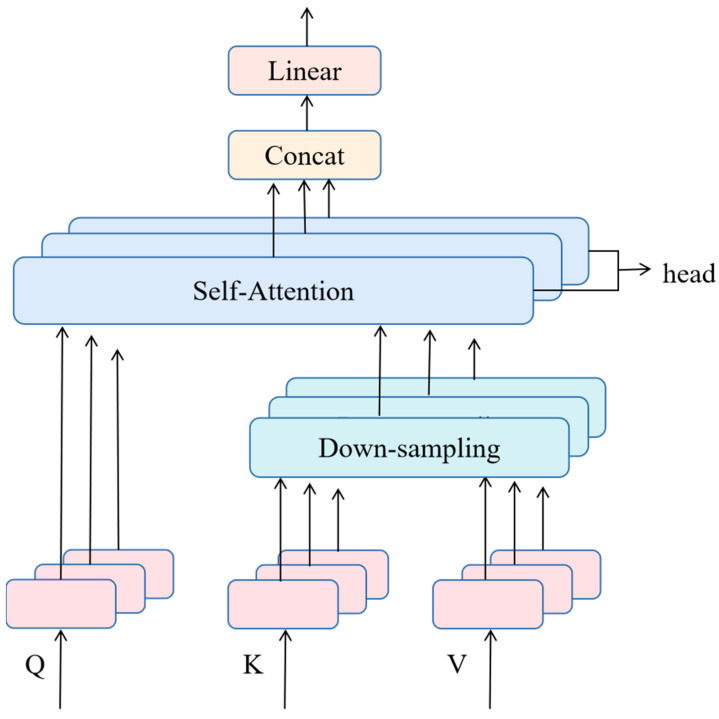
The architecture of the modified multi-head self-attention.

The transformer has gained significant attention in computer vision due to its successful applications. Specifically, the self-attention mechanism, which is a key component of the transformer, is capable of capturing long-range dependencies, making it an attractive technique. In this paper, an M-MHSA network is introduced, where *K* and *V* are projected to a low-dimensional embedding using a lightweight down-sampling. This operation reduces the computing burden caused by performing attention calculations on all pixels, while simultaneously enriches feature subspace’s diversity by independent attention heads. Moreover, it assigns weights based on the inter-pixel correlations, allowing for the extraction of global feature dependency and overcoming the limitation of the small receptive field of a traditional CNN. The network architecture of M-MHSA is shown in Figure 2.

Hyperspectral pixels can be viewed as a sequence of vectors $X\in R^{(H\times W)\times B}$. Each vector is multiplied by three weight matrices to obtain Query (*Q*), Key (*K*), and Value (*V*). The linear transformation for this process can be expressed as follows:

(2) $$\begin{array}{l} Q=W_{q}X\\ K=W_{k}X\\ V=W_{v}X \end{array}$$

where $W_{q}$, $W_{k}$, and $W_{v}$ represent the transformation matrix of *Q*, *K*, and *V*, respectively. 

The attention weight calculation can be expressed as:

(3) $$Attention\left(Q,K,V\right)=soft\max\left(\frac{QK^{Τ}}{\sqrt{d_{k}}}\right)V$$

where d_k_ represents the dimension of *Q* and *K*.

To focus on different parts of the feature representation and extract richer long-range dependencies, *Q*, *K*, and *V* are divided into *h* submatrix as follows:

(4) $$\begin{array}{l} Q=\left\{Q_{1},Q_{2},\ldots ,Q_{i},\ldots ,Q_{h}\right\}\\ K=\left\{K_{1},K_{2},\ldots ,K_{i},\ldots ,K_{h}\right\}\\ V=\left\{V_{1},V_{2},\ldots ,V_{i},\ldots ,V_{h}\right\} \end{array}$$

where h represents the number of heads.

The *i-th* head can be expressed as:

(5) $${head}_{i}=Attention\left(Q_{i},K_{i},V_{i}\right)$$

where $Q_{i},\;K_{i},\;V_{i}\in R^{(H\times W)\times \frac{B}{h}}$.

Multiple independent heads are spliced together to form MHSA, so MHSA can be expressed as:

(6) $$MHSA\left(Q,K,V\right)=concat\left(head_{1},\ldots ,head_{h}\right)W^{O}$$

where $W^{O}$ indicates the output projection matrix. 

To reduce the computational burden caused by dot product of *Q* and *K*, we propose to perform down-sampling on *K* and *V* after obtaining them, while preserving important information. Specifically, we reduce the spatial dimensions of *K* and *V* from (*H × W*) to (16 × 16), which $K_{i},V_{i}\in R^{(16\times 16)\times \frac{B}{h}}$ in each head. This not only reduces the computational cost but also enables the network to capture long-range dependencies of the input image pixels. The modified MHSA can be expressed as:

(7) M−MHSA=MHSA(Q,DSA(K,V))

where DSA(·) function represents a down-sampling operation.

### 2.3. Spectral-Coordinate Attention Fusion Network (SCA) 

HSIs typically contain hundreds of bands, but many of them contribute little to the HSI classification and thus lead to poor classification performance. In this work, we perform spectral attention and coordinate attention for better utilization of the discriminative spectral and spatial features present in HSIs. Finally, we perform feature fusion to further enhance the HSI classification performance.

#### 2.3.1. Spectral Attention 

**Figure 3:**
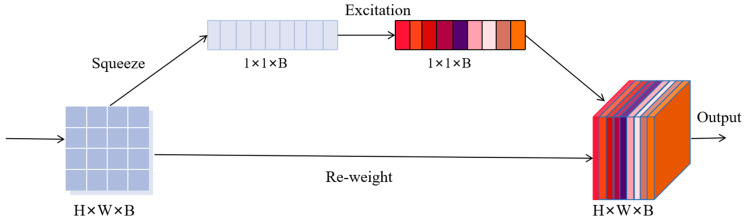
The architecture of spectral attention module.

As shown in Figure 3, we incorporate the SE-Net architecture to recalibrate the spectral features in the HSI to strengthen the connections between spectral bands. This helps the network focus on valuable spectral channel information while suppressing irrelevant or invalid characteristic channel information.

Let $X=[x_{1},x_{2},\ldots ,x_{B}]\in R^{H\times W\times B}$ represents the input of SE network and $x_{b}\in R^{H\times W}$ represents *b-th* channel of feature mapping. By using a squeeze operation *F_sq_*, the input feature map can be compressed along the spatial dimension, reducing two-dimensional features to one-dimensional data. This is achieved through global average pooling. $z_{b}\in R^{B}$ generated by squeeze can be expressed as follows:

(8) $$z_{b}=F_{sq}\left(x_{b}\right)=\frac{1}{H\times W}\sum_{i=1}^{H}\sum_{j=1}^{W}x_{b}\left(i,j\right)$$

This operation is equivalent to indicating the value distribution of b feature maps. $x_{b}\left(i,j\right)$ represents the element value of the *b-th* feature map at position (*i*, *j*). 

The two fully connected layer networks are utilized to automatically learn the interdependency between different channels, with the importance of each channel determined by learned weight coefficients *W_E_*. This enables the Excitation formula to capture the dependency relationship between channels, which can be expressed as follows:

(9) $$s=F_{ex}\left(z,W_{E}\right)=\sigma \left(g\left({z,W}_{E}\right)\right)=\sigma \left(W_{2}\delta \left(W_{1}z\right)\right)$$

where s represents the weight of each feature map, δ is the ReLU activation function operation, $W_{1}\in R^{\frac{B}{r}\times B}$, $W_{2}\in R^{B\times \frac{B}{r}}$, and r represents a ratio of dimension reduction.

At last, the output of the SE block is obtained by rescaling *X* with the activations s can be expressed as:

(10) $${\tilde{x}}_{b}=F_{scale}\left(x_{b},s_{b}\right)=s_{b}\cdot x_{b}$$

where $\tilde{X}=[{\tilde{x}}_{1},{\tilde{x}}_{2},\ldots ,{\tilde{x}}_{B}]$, $F_{scale}(x_{b},s_{b})$ represents channel-wise scalar multiplication between the scalar $s_{b}$ and feature mapping $x_{b}$.

#### 2.3.2. Coordinate Attention

**Figure 4:**
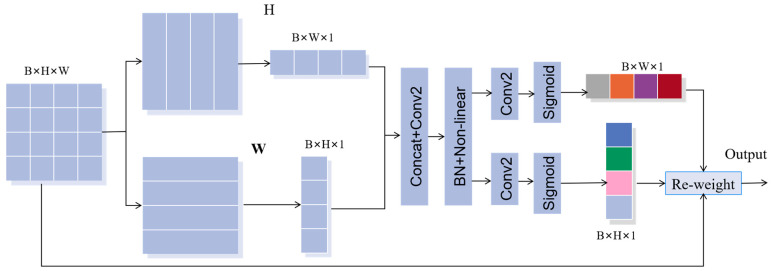
The architecture of the coordinate attention.

SE module uses 2-D global pooling to weigh channels and capture dependencies between them, providing significant performance gains at a relatively low computational cost. However, the SE module only considers information encoding between channels and ignores the importance of positional information, which is actually crucial for obtaining target information. Therefore, we propose incorporating Coordinate Attention (CA) to the network, which not only captures cross-channel information but also provides information on direction and position perception, enabling the model to locate and identify the target of interest more accurately. Moreover, the CA module is flexible and lightweight, making it easy to integrate into classic modules. The CA module encodes channel relationships and long-range dependencies through precise location information, similar to the SE module. It consists of two steps: coordinate information embedding and coordinate attention generation. By incorporating the CA module, we can improve the accuracy of the model in identifying targets, while still maintaining computational efficiency. The structure of CA is shown in Figure 4. 

First, the input $X=[x_{1},x_{2},\ldots ,x_{B}]\in R^{H\times W\times B}$ is processed by the CA module, which converts it into two separate vectors using two-dimension global pooling. This operation encodes each channel along the two spatial directions using average pooling cores of sizes (*H*, 1) and (1, *W*), respectively. 

The output of *b*-channel at height *H* can be expressed as:

(11) $$z_{b}^{h}\left(h\right)=\frac{1}{W}\sum_{0\leq i\leq W}x_{b}\left(h,i\right)$$

Similarly, the output of channel *b* at width *W* can be expressed as:

(12) $$z_{b}^{w}\left(w\right)=\frac{1}{H}\sum_{0\leq j\leq H}x_{b}\left(j,w\right)$$

After the two transforms are generated, feature aggregation is carried out along two spatial directions. The two transformed vectors are concatenated and passed through the 1 × 1 convolution transformation function $F_{1}$ to generate an intermediate feature map $f\in R^{B/r\times \left(H+W\right)}$, which captures the spatial information of the horizontal and vertical directions. The parameter r represents the reduction ratio, and the function f can be expressed as:

(13) $$f=\delta \left(F_{1}\left(\left[z^{h},z^{w}\right]\right)\right)$$

Next, we divide the function f into two separate tensors $f^{h}\in R^{B/r\times H}$ and $f^{w}\in R^{B/r\times W}$ along the two spatial directions. The resulting feature maps are then transformed using two 1 × 1 2-D convolution operations, enabling them to be brought to the same channel number as the original input *X*; the formula is as follows:

(14) $$\begin{array}{l} o^{h}=\sigma \left(F_{h}\left(f^{h}\right)\right)\\ o^{w}=\sigma \left(F_{w}\left(f^{w}\right)\right) \end{array}$$

where σ is the sigmoid function. And then, $o^{h}$ and $o^{w}$ are then expanded and used as the attention weights of the *H* and *W* direction, respectively. The final output of the coordinate attention module can be defined as:

(15) $$y_{b}\left(i,j\right)=x_{b}\left(i,j\right)\times o_{b}^{h}\left(i\right)\times o_{b}^{w}\left(j\right)$$

## 3. Experiments

In this section, we conduct experiments on three classical public datasets: the Indian Pines, the Pavia University, and the Salinas datasets to evaluate the performance of our proposed method. We compare our method with several existing methods, including SVM [8], FDSSC [21], SSRN [20], HybridSN [31], CGCNN [32], DBMA [33], and DBDA [29]. We evaluate the effectiveness of our proposed method using overall accuracy (OA), average accuracy (AA), and Kappa statistics (KPP). OA measures the overall accuracy of a classification model, which is defined as the proportion of correctly classified samples in the entire test set. AA is the average accuracy per class, which considers the accuracy of the model for each class. Kappa index is a measure of agreement between the predicted and true class labels that considers the agreement that could occur by chance. The kappa index can be calculated from the confusion matrix, and it is widely used in multi-class classification problems to evaluate the performance of a classifier.

### 3.1. Configuration for Parameters

The proposed MSSCA method comprises of four modules: PCN, M-MHSA, SA, and CA. Specifically, the PCN module utilizes two network layers and 128 1×1 convolution kernels, and the activation functions used in PCN are leaky rectified linear units (Leaky ReLUs). In the M-MHSA, the numbers of the heads are set to four, and we reduce the spatial dimensions of *K* and *V* from (*H×W*) to (*16×16*). We adopt a learning rate of 0.005 for iterative updating, and the maximum number of iterations is set to 600. Finally, we conduct experiments on an NVIDIA Geforce RTX 3090 computer with 16 GB of RAM. The experiments were carried out on a Windows 10 Home Edition platform, and the code was implemented using Python 3.7.13 and PyTorch 1.11.0.

### 3.2. HSI Datasets

**Figure 5:**
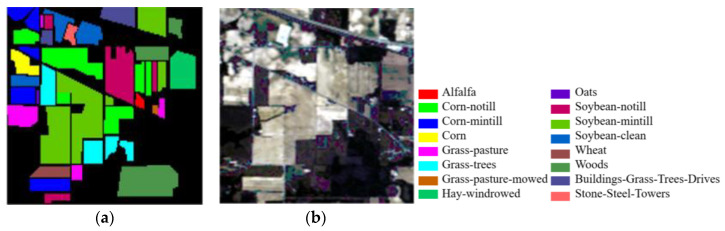
Indian Pines images: (**a**) false-color image; (**b**) ground truth.

**Figure 6:**
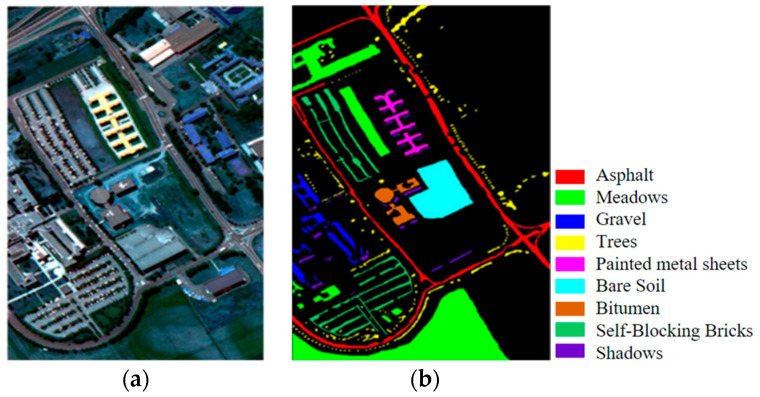
Pavia University images: (**a**) false-color image; (**b**) ground truth.

**Figure 7:**
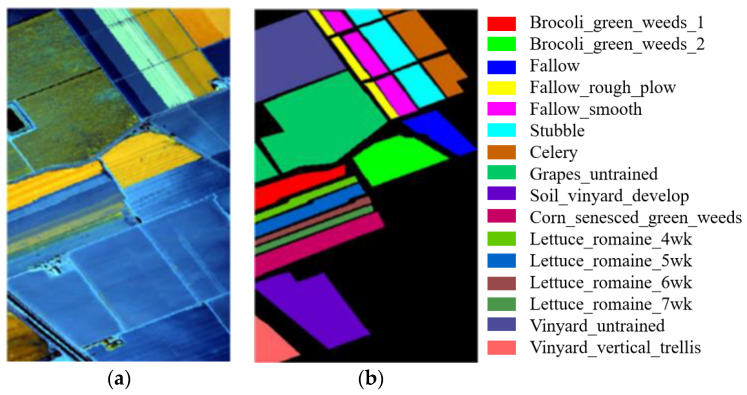
Salinas images: (**a**) false-color image; (**b**) ground truth.

(1) Indian Pines dataset: The first dataset is the Indian Pines dataset acquired by the imaging spectrometer AVIRIS in northwest Indiana, USA. The HSI of this scene consists of 145 × 145 pixels, with 220 bands and a spatial resolution of 20 m/pixel. After removing interference bands, the dataset includes 200 available bands. The dataset comprises 16 different categories of ground objects, with 10,249 reference samples. For training, validation, and testing purposes, 10%, 1%, and 89% of each category were randomly selected, respectively. Figure 5 displays the false-color image and real map, while Table 1 provides detailed category information for this HSI dataset.

(2) Pavia University dataset: The second dataset is the Pavia University dataset acquired at the Pavia University using the Imaging Spectrometer Sensor ROSIS of the Reflexology System. The HSI of this scene comprises 610 × 340 pixels, with 115 bands and a spatial resolution of 1.3 m/pixel. After removing the interference bands, the dataset includes 103 available bands. The dataset contains nine different categories of ground objects, with 42,776 reference samples. For training, verification, and testing purposes, 1%, 1%, and 98% of each category’s samples were randomly selected, respectively. Figure 6 displays the false-color image and real map, while Table 2 provides detailed class information for this HSI dataset.
(3) Salinas dataset: The third dataset is the Salinas dataset acquired by the AVIRIS Imaging Spectrometer sensor over the Salinas Valley. The HSI of the scene comprises 512 × 217 pixels, with 224 bands and a spatial resolution of 3.7 m/pixel. After discarding 20 interference bands, the dataset includes 204 available bands. The dataset contains 16 different categories of features, with 54,129 samples available for the experiment. For training, verification, and testing purposes, 1%, 1%, and 98% of each category’s samples were randomly selected, respectively. Figure 7 displays the false-color image and the real object map, while Table 3 provides detailed class information for this HSI dataset.

**Table 1:** Category information of Indian Pines Dataset.

No.	Class	Train.	Val.	Test.
1	Alfalfa	5	1	48
2	Corn-notill	143	14	1277
3	Corn-mintill	83	8	743
4	Corn	23	2	209
5	Grass-pasture	49	4	444
6	Grass-trees	74	7	666
7	Grass-pasture-mowed	2	1	23
8	Hay-windrowed	48	4	437
9	Oats	2	1	17
10	Soybean-notill	96	9	863
11	Soybean-mintill	246	24	2198
12	Soybean-clean	61	6	547
13	Wheat	21	2	189
14	Woods	129	12	1153
15	Buildings-Grass-Trees-Drives	38	3	339
16	Stone-Steel-Towers	9	1	85
	Total	1029	99	9238

**Table 2:** Category information of Pavia University dataset.

No.	Class	Train.	Val.	Test.
1	Asphalt	67	67	6497
2	Meadows	187	187	18,275
3	Gravel	21	21	2057
4	Trees	31	31	3002
5	Painted metal sheets	14	14	1317
6	Bare Soil	51	51	4927
7	Bitumen	14	14	1302
8	Self-Blocking Bricks	37	37	3608
9	Shadows	10	10	927
	Total	432	432	41,912

**Table 3:** Category information of Salinas dataset.

No.	Class	Train.	Val.	Test.
1	Brocoli_green_weeds_1	21	21	1967
2	Brocoli_green_weeds_2	38	38	3650
3	Fallow	20	20	1936
4	Fallow_rough_plow	14	14	1366
5	Fallow_smooth	27	27	2624
6	Stubble	40	40	3879
7	Celery	36	36	3507
8	Grapes_untrained	113	113	11,045
9	Soil_vinyard_develop	63	63	6077
10	Corn_senesced_green_weeds	33	33	3212
11	Lettuce_romaine_4 wk	11	11	1046
12	Lettuce_romaine_5 wk	20	20	1887
13	Lettuce_romaine_6 wk	10	10	896
14	Lettuce_romaine_7 wk	11	11	1048
15	Vinyard_untrained	73	73	7122
16	Vinyard_vertical_trellis	19	19	1769
	Total	549	549	53,031

### 3.3. Comparison of Classification Results

**Figure 8:**
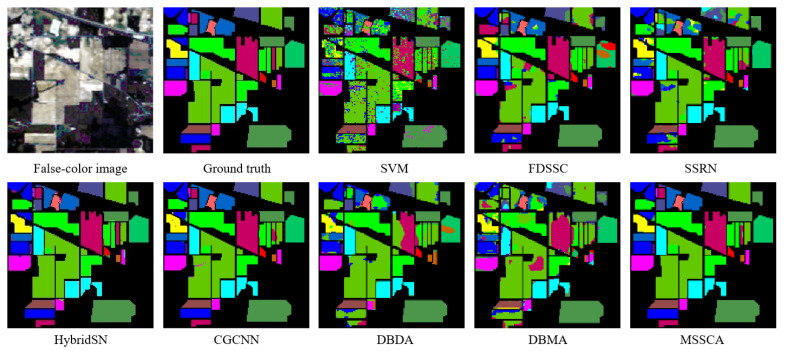
Classification maps of different methods for the Indian Pines dataset.

**Figure 9:**
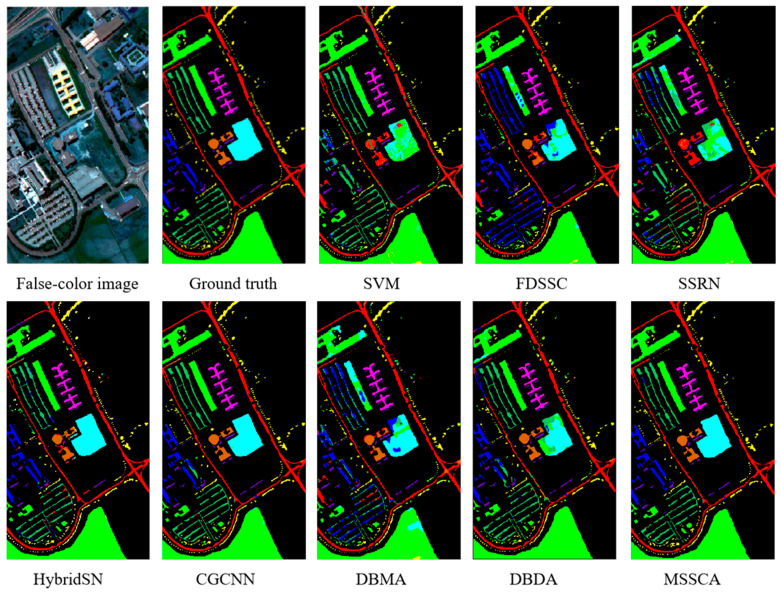
Classification maps of different methods for the Pavia University dataset.

**Figure 10:**
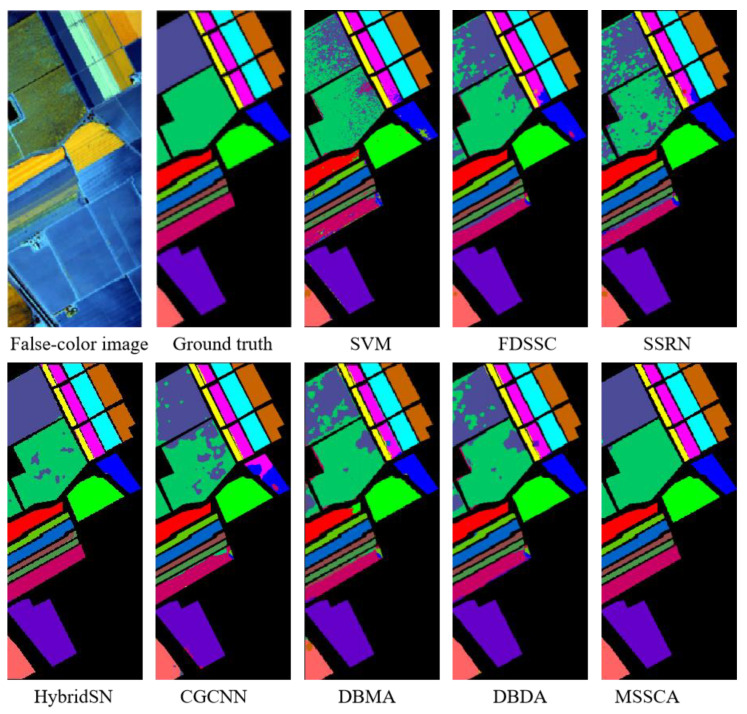
Classification maps of different methods for the Salinas dataset.

In this section, we evaluate the performance of our proposed method and compare it with several deep learning-based networks on three datasets. We conducted 10 repeated experiments and report the experimental results as mean ± standard deviation. The classification accuracy of different classification methods on each dataset is presented in Table 4, Table 5 and Table 6. Additionally, we display the classification maps obtained by these methods in Figure 8, Figure 9 and Figure 10.

Experiments on the Indian Pines dataset demonstrate that our proposed method achieves the highest classification accuracy compared to other methods. The SSRN network extracts spectral and spatial features through continuous spectral and spatial residual blocks, respectively, effectively alleviating the gradient descent phenomenon. Compared to traditional methods, it has shown significant improvement. Our proposed method further improves the accuracy by incorporating an attention mechanism, which has been shown to be more effective than that of SSRN. As shown in Table 4, the proposed method improves the overall accuracy by 25.84% and 15.10% compared to DBMA and DBDA, respectively. Moreover, it also surpasses the advanced CNN network CGCNN.

As shown in Figure 8, our proposed method has fewer misclassification points, which is more consistent with the ground truth. In contrast, the traditional SVM method produces a lot of salt and pepper noise, resulting in many misclassifications. By combining spectral and coordinate attention, our network focuses on effective information, resulting in a significant reduction in the error rate and smoother classification maps.

Similar to the results on the Indian Pines dataset, our proposed method achieves the best classification results on the Pavia University dataset compared to other methods, demonstrating the stability of our network. As shown in Table 5, our proposed method outperforms current state-of-the-art methods, such as CGCNN, DBMA, and DBDA, by improving OA by 1.05%, 15.81%, and 7.16%, respectively. Moreover, our proposed MSSCA method achieves an accuracy of 95% in each category, indicating its effectiveness.

Figure 9 shows that our proposed MSSCA method has fewer misclassification points on the Pavia University dataset, which is more consistent with the ground truth compared to CGCNN, which has shown good performance on this dataset. 

Table 6 presents the classification results on the Salinas dataset, where our proposed MSSCA method achieves the best overall accuracy (OA), average accuracy (AA), and Kappa statistics (KPP), with an OA accuracy of 99.41%. Moreover, our proposed method achieves almost the best classification results in each category.

The classification results of different methods on the Salinas dataset are shown in Figure 10, where our proposed MSSCA method outperforms other methods in misclassified categories, such as Lettuce_romaine_7 wk and Vinyard_untrained. The classification map generated by our method is more consistent with the ground truth, and the class boundaries are clearer.

### 3.4. Ablation Study

To evaluate the effectiveness of each module in the MSSCA architecture, we conducted a set of ablation experiments by splitting and combining different network modules. Table 7 presents the classification accuracy of different modules. As can be seen from the table, using only the SE or CA module results in lower OA compared to when both modules are combined. This indicates that the addition of both SE and CA modules improves the classification accuracy. The SE module focuses on the importance of channels, while the CA module focuses on the importance of spatial locations. By paying attention to both channel and coordinate information, the model can more effectively utilize relevant information, resulting in improved classification results. Moreover, incorporating the PCN module improves classification accuracy by providing more discriminative input and optimizing network feature modules.

### 3.5. Training Sample Ratio 

**Figure 11:**
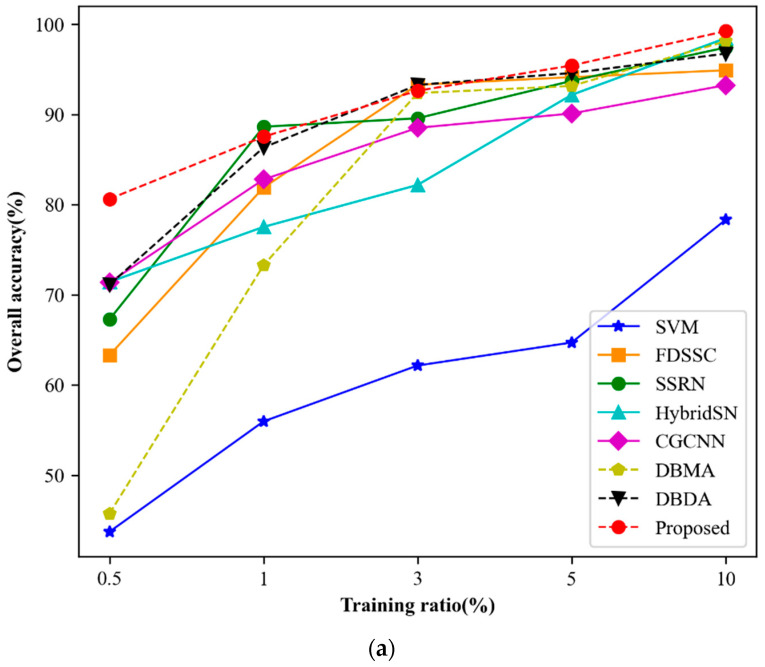

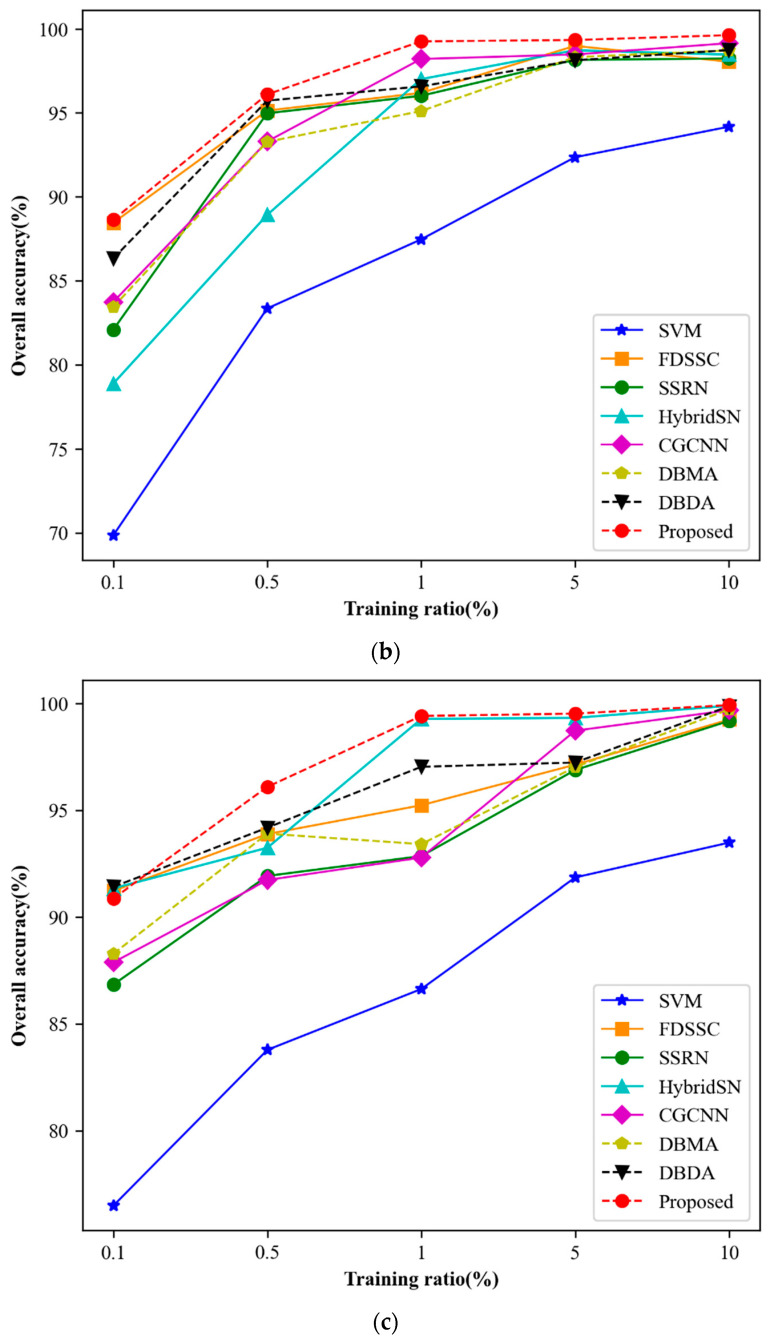
The OA of different methods with varying ratios of training samples. (**a**) Indian Pines. (**b**) Pavia University. (**c**) Salinas.

As is well known, deep learning algorithms heavily depend on large amounts of high-quality labeled data, and the network performance improves as the quantity of labeled data increases. In this section, we analyze the comparative results of different training ratios. Figure 11 presents the experimental results. For the Indian Pines dataset, we use 0.5%, 1%, 3%, 5%, and 10% samples as the training sets. For PU and SV datasets, we use 0.1%, 0.5%, 1%, 5%, and 10%, respectively.

As shown in Figure 11a–c, the classification accuracy of all three datasets increases as the training ratio increases. With sufficient training samples, almost perfect classification results can be achieved. Moreover, as the training ratio increases, the difference in classification accuracy between different methods becomes smaller. Notably, even with a small training ratio, our proposed MSSCA method outperforms other comparison methods. The performance of our proposed method exhibits a steady growth trend across all three datasets, indicating its effectiveness and stability.

### 3.6. Running Time

This section presents the training and testing times of different methods on different datasets, as shown in Table 8, Table 9 and Table 10. Since the goal of HSI classification is to assign a specific label to each pixel, we consider the time taken to classify all pixels as the test time. From the tables, we can see that SVM has a short training time, but it can only extract shallow features and has poor classification performance. Existing deep learning methods such as DBMA and DBDA perform well but have long testing times. In contrast, our proposed MSSCA method not only achieves outstanding classification performance, but also has a short testing time and low computational cost. This is because we use a lightweight attention mechanism, which reduces the computational cost while improving performance.

## 4. Conclusions

In this paper, we propose an effective deep learning method called MSSCA for HSI classification. In MSSCA, to reduce the computational burden caused by the dot-product operation, the down-sampling operation is introduced into MHSA, and the novel M-MHSA is proposed to depict the long-range dependencies of HSI pixels. On this basis, we integrate SE and CA networks to effectively leverage spectral and spatial coordinate information, which enhances network performance and classification results without compromising network complexity or computational costs. Three classical datasets, including Indian Pines, Pavia University, and Salinas, are used to evaluate the proposed method. The proposed method’s performance was validated by a performance comparison with some classical methods, such as SSRN, HybridSN, and DBDA. The proposed MSSCA method achieved an overall accuracy of 99.96% for Indian Pines datasets, 99.26% for Pavia University datasets, and 99.41% for Salinas datasets, outperforming most existing HSI classification methods, highlighting the effectiveness and efficiency of our proposed method in HSI classification. In the future, we will continue to explore more lightweight and effective classification frameworks to HSI classification under complex conditions.

## Figures and Tables

**Table 4:** Classification performance of different methods on the Indian Pines dataset.

Class	SVM	FDSSC	SSRN	HybridSN	CGCNN	DBMA	DBDA	MSSCA
1	18.88 ± 7.76	45.20 ± 30.35	75.95 ± 29.99	97.16 ± 2.27	97.04 ± 2.46	39.23 ± 19.48	73.37 ± 25.28	**97.54 ± 1.58**
2	46.05 ± 6.40	78.02 ± 12.10	85.75 ± 4.76	97.15 ± 0.56	98.62 ± 0.59	70.87 ± 10.59	79.10 ± 8.95	**99.01 ± 0.67**
3	45.88 ± 15.31	75.69 ± 14.23	83.49 ± 12.87	98.25 ± 0.65	98.61 ± 1.13	67.69 ± 14.74	79.24 ± 12.45	**99.35 ± 0.62**
4	30.05 ± 7.38	74.05 ± 30.61	77.95 ± 27.61	97.90 ± 2.75	97.91 ± 1.32	64.24 ± 20.66	82.49 ± 16.99	**98.86 ± 1.06**
5	71.42 ± 21.58	96.71 ± 3.60	96.07 ± 7.65	98.49 ± 0.96	98.64 ± 1.31	89.66 ± 6.55	96.89 ± 3.97	**99.44 ± 0.78**
6	74.53 ± 4.01	90.74 ± 12.06	94.83 ± 4.15	98.92 ± 0.38	99.75 ± 0.16	85.52 ± 4.97	95.36 ± 5.63	**99.75 ± 0.18**
7	25.70 ± 15.00	36.11 ± 28.85	70.04 ± 29.66	**100.00 ± 0.00**	99.20 ± 0.16	32.95 ± 26.11	30.56 ± 14.81	**100.00 ± 0.00**
8	87.20 ± 3.06	97.55 ± 5.29	97.82 ± 3.43	99.67 ± 0.31	99.63 ± 0.48	99.03 ± 2.20	**100.00 ± 0.00**	99.95 ± 0.10
9	18.28 ± 9.91	43.79 ± 29.36	79.96 ± 28.87	92.38 ± 5.25	**100.00 ± 0.00**	12.05 ± 5.46	45.58 ± 17.69	86.67 ± 15.15
10	50.16 ± 8.78	80.41 ± 12.34	87.96 ± 7.00	**98.74 ± 0.81**	97.29 ± 1.48	70.97 ± 13.57	84.06 ± 8.54	97.89 ± 1.26
11	52.03 ± 4.62	80.90 ± 8.31	86.75 ± 6.39	99.16 ± 0.26	99.22 ± 0.46	73.04 ± 5.70	83.82 ± 9.71	**99.49 ± 0.49**
12	34.82 ± 10.77	74.71 ± 27.55	85.83 ± 5.92	97.47 ± 1.17	97.95 ± 0.91	63.04 ± 15.42	81.08 ± 12.23	**98.94 ± 0.93**
13	76.72 ± 5.64	92.72 ± 12.56	99.50 ± 1.49	98.02 ± 1.64	99.45 ± 0.01	92.24 ± 7.71	93.20 ± 8.57	**99.78 ± 0.44**
14	79.21 ± 5.25	91.99 ± 4.60	93.93 ± 3.81	99.32 ± 0.44	99.80 ± 0.13	93.12 ± 4.56	93.90 ± 4.10	**99.96 ± 0.04**
15	48.80 ± 20.15	69.33 ± 35.62	93.06 ± 4.79	97.64 ± 1.58	98.01 ± 0.02	67.51 ± 12.90	89.00 ± 13.52	**98.64 ± 1.87**
16	98.50 ± 2.57	80.07 ± 7.38	95.68 ± 3.51	91.02 ± 4.07	**99.04 ± 0.12**	81.27 ± 12.08	83.83 ± 6,76	97.10 ± 2.36
OA (%)	55.98 ± 2.75	81.89 ± 6.27	88.63 ± 3.98	98.44 ± 0.16	98.85 ± 0.18	73.39 ± 2.88	84.13 ± 1.19	**99.23 ± 0.19**
AA (%)	53.64 ± 3.45	76.00 ± 11.45	87.79 ± 8.56	97.58 ± 0.82	**98.76 ± 0.18**	68.91 ± 4.26	80.72 ± 4.33	98.27 ± 1.03
KPP (×100)	48.72 ± 3.32	79.19 ± 7.37	86.97 ± 4.63	98.23 ± 0.18	98.69 ± 0.21	69.53 ± 3.27	81.85 ± 1.39	**99.12 ± 0.21**

**Table 5:** Classification performance of different methods on the Pavia University dataset.

Class	SVM	FDSSC	SSRN	HybridSN	CGCNN	DBMA	DBDA	MSSCA
1	85.83 ± 7.24	91.37 ± 5.38	91.80 ± 7.91	95.13 ± 1.81	98.49 ± 0.81	91.41 ± 2.88	93.76 ± 2.83	**99.10 ± 0.60**
2	73.97 ± 4.12	94.37 ± 4.01	86.40 ± 4.03	99.16 ± 0.49	98.92 ± 0.40	89.02 ± 5.77	96.20 ± 2.11	**99.96 ± 0.03**
3	31.14 ± 8.49	59.20 ± 18.76	59.59 ± 20.11	88.73 ± 4.90	87.73 ± 5.44	65.63 ± 23.57	81.67 ± 9.26	**95.76 ± 2.94**
4	70.16 ± 25.62	97.69 ± 1.72	98.38 ± 3.37	98.18 ± 0.77	97.11 ± 1.26	94.38 ± 4.47	**98.22 ± 1.39**	97.23 ± 1.62
5	97.27 ± 2.47	99.40 ± 0.75	98.76 ± 1.58	98.98 ± 0.93	**100.00 ± 0.00**	99.44 ± 0.94	98.17 ± 2.50	99.95 ± 0.09
6	45.73 ± 22.56	86.06 ± 9.10	77.77 ± 8.00	98.66 ± 0.96	99.55 ± 0.69	74.11 ± 11.22	90.50 ± 8.77	**99.95 ± 0.07**
7	43.20 ± 7.05	90.68 ± 7.72	65.61 ± 25.35	96.64 ± 2.37	99.11 ± 0.64	66.72 ± 14.16	85.39 ± 15.01	**99.85 ± 0.27**
8	64.45 ± 9.43	68.03 ± 20.33	74.50 ± 14.25	90.69 ± 2.72	97.77 ± 1.93	66.76 ± 16.60	79.61 ± 9.58	**98.13 ± 1.98**
9	**99.90 ± 0.11**	96.91 ± 1.91	98.28 ± 1.61	97.21 ± 1.86	99.98 ± 0.04	90.27 ± 11.33	94.35 ± 3.64	99.74 ± 0.16
OA (%)	69.86 ± 2.21	88.46 ± 4.24	82.10 ± 3.01	97.01 ± 0.69	98.21 ± 0.13	83.45 ± 3.58	92.10 ± 1.12	**99.26 ± 0.18**
AA (%)	67.96 ± 5.27	87.08 ± 4.55	83.45 ± 2.55	95.93 ± 0.87	97.63 ± 0.26	81.97 ± 4.60	90.88 ± 1.41	**98.85 ± 0.30**
KPP (×100)	58.26 ± 3.78	84.61 ± 5.83	75.88 ± 4.07	96.02 ± 0.92	97.63 ± 0.18	77.85 ± 4.93	89.52 ± 1.45	**99.02 ± 0.24**

**Table 6:** Classification performance of different methods on the Salinas dataset.

Class	SVM	FDSSC	SSRN	HybridSN	CGCNN	DBMA	DBDA	MSSCA
1	92.70 ± 7.26	96.81 ± 9.56	96.51 ± 6.29	99.79 ± 0.24	99.97 ± 0.04	97.16 ± 8.39	95.67 ± 8.61	**99.98 ± 0.02**
2	98.61 ± 1.01	99.89 ± 0.29	92.61 ± 12.10	99.97 ± 0.02	99.12 ± 0.82	98.48 ± 2.16	99.99 ± 0.02	**100.00 ± 0.00**
3	75.17 ± 7.04	93.61 ± 4.44	92.84 ± 7.88	99.96 ± 0.04	66.86 ± 3.93	95.16 ± 2.74	97.65 ± 1.25	**100.00 ± 0.00**
4	96.79 ± 0.99	95.65 ± 3.38	95.55 ± 3.39	98.35 ± 1.05	99.79 ± 0.18	85.27 ± 4.98	90.16 ± 3.58	**99.88 ± 0.12**
5	91.04 ± 5.51	95.99 ± 6.43	89.26 ± 8.50	**99.93 ± 0.07**	95.67 ± 4.80	94.43 ± 6.80	92.90 ± 6.88	98.28 ± 1.81
6	99.87 ± 0.28	99.99 ± 1.62	99.91 ± 0.15	99.93 ± 0.10	99.76 ± 0.30	99.23 ± 1.14	99.88 ± 0.23	**99.96 ± 0.07**
7	94.30 ± 2.30	99.27 ± 0.84	98.84 ± 2.19	**100.00 ± 0.00**	99.91 ± 0.05	95.84 ± 5.05	99.71 ± 0.21	99.98 ± 0.03
8	65.65 ± 3.55	84.04 ± 6.33	76.61 ± 8.99	98.81 ± 0.88	91.43 ± 3.59	81.52 ± 8.59	81.60 ± 9.69	**99.12 ± 0.81**
9	95.03 ± 6.16	98.88 ± 0.76	98.73 ± 1.33	99.96 ± 0.02	99.48 ± 0.31	98.53 ± 1.52	97.80 ± 1.98	**100.00 ± 0.00**
10	80.87 ± 11.45	95.96 ± 2.68	94.79 ± 3.45	**98.96 ± 1.06**	93.76 ± 2.90	92.15 ± 5.03	94.29 ± 2.99	97.03 ± 2.46
11	58.82 ± 27.59	**100.00 ± 0.00**	93.23 ± 4.40	99.21 ± 1.05	97.50 ± 2.17	80.78 ± 17.94	93.45 ± 4.67	99.87 ± 0.18
12	86.41 ± 10.28	99.00 ± 1.35	94.51 ± 7.94	99.81 ± 0.33	99.82 ± 0.31	97.75 ± 2.10	98.56 ± 1.31	**100.00 ± 0.00**
13	81.66 ± 11.84	98.24 ± 2.60	92.66 ± 7.85	98.77 ± 2.28	98.28 ± 1.79	86.76 ± 16.37	99.53 ± 0.24	**99.93 ± 0.09**
14	80.08 ± 14.32	94.24 ± 4.79	97.01 ± 1.50	**99.60 ± 0.45**	98.10 ± 2.05	89.69 ± 7.44	95.76 ± 1.86	98.70 ± 0.83
15	48.14 ± 24.59	77.43 ± 9.62	69.53 ± 10.99	97.88 ± 2.67	75.90 ± 12.45	75.30 ± 10.42	80.91 ± 5.96	**99.33 ± 0.41**
16	88.65 ± 15.52	99.66 ± 0.69	99.02 ± 1.39	**100.00 ± 0.00**	96.12 ± 0.64	96.39 ± 5.84	99.11 ± 1.73	99.55 ± 0.55
OA (%)	80.50 ± 2.68	91.23 ± 1.94	86.85 ± 1.98	99.27 ± 0.29	92.78 ± 1.20	88.29 ± 2.03	91.41 ± 2.86	**99.41 ± 0.32**
AA (%)	83.36 ± 5.23	94.77 ± 1.43	92.60 ± 1.20	99.43 ± 0.19	94.47 ± 0.56	91.53 ± 2.09	94.81 ± 0.91	**99.48 ± 0.25**
KPP (×100)	78.21 ± 3.06	90.23 ± 2.18	85.33 ± 2.20	99.19 ± 0.32	91.94 ± 1.36	86.96 ± 2.29	90.41 ± 3.22	**99.34 ± 0.35**

**Table 7:** Ablation study on attention modules (OA%).

Dataset	CA	SE	CA + SE	PCN + SE + CA
**Indian Pines**	98.19 ± 0.17	98.18 ± 0.84	99.15 ± 0.13	**99.23 ± 0.19**
**Pavia University**	98.09 ± 0.40	98.17 ± 0.24	98.52 ± 0.18	**99.26 ± 0.18**
**Salinas**	97.79 ± 0.26	98.20 ± 0.73	98.60 ± 0.47	**99.41 ± 0.32**

**Table 8:** Running time (s) of different methods on the Indian Pines dataset.

Dataset	Methods	Train Time	Test Time
**Indian Pines**	SVM	**44.50**	15.43
	FDSSC	129.39	205.27
	SSRN	77.23	204.72
	HybridSN	239.82	6.89
	CGCNN	108.23	1.72
	DBMA	107.87	59.60
	DBDA	100.107	28.35
	MSSCA	52.93	**0.52**

**Table 9:** Running time (s) of different methods on the Pavia University dataset.

Dataset	Methods	Train Time	Test Time
**Pavia University**	SVM	**16.42**	53.05
	FDSSC	81.14	171.25
	SSRN	132.98	10.11
	HybridSN	97.12	52.60
	CGCNN	679.44	6.69
	DBMA	146.28	201.41
	DBDA	58.81	115.80
	MSSCA	99.65	**8.93**

**Table 10:** Running time (s) of different methods on the Salinas dataset.

Dataset	Methods	Train Time	Test Time
**Salinas**	SVM	**9.85**	3.82
	FDSSC	129.39	205.27
	SSRN	77.23	204.72
	HybridSN	375.15	46.48
	CGCNN	340.81	4.69
	DBMA	84.12	323.03
	DBDA	62.29	161.02
	MSSCA	69.49	**3.41**

## Data Availability

All data used in this paper are available at https://ehu.eus/ccwintco/index.php/Hyperspectral_Remote_Sensing_Scenes, accessed on 10 April 2022.
